# A Review of Neoadjuvant Therapy and the Watch-and-Wait Protocol in Rectal Cancer: Current Evidence and Future Directions

**DOI:** 10.7759/cureus.68461

**Published:** 2024-09-02

**Authors:** Iulian M Slavu, Octavian Munteanu, Florin Filipoiu, Raluca Tulin, Anca Monica Macovei Oprescu, Ileana Dima, Iulian A Dogaru, Adrian Tulin

**Affiliations:** 1 Anatomy, Carol Davila University of Medicine and Pharmacy, Bucharest, ROU; 2 Embryology, Carol Davila University of Medicine and Pharmacy, Bucharest, ROU; 3 Endocrinology, Agrippa Ionescu Emergency Clinical Hospital, Bucharest, ROU; 4 Gastroenterology, Agrippa Ionescu Emergency Clinical Hospital, Bucharest, ROU; 5 General Surgery, Agrippa Ionescu Emergency Clinical Hospital, Bucharest, ROU; 6 Medicine, Carol Davila University of Medicine and Pharmacy, Bucharest, ROU

**Keywords:** radiotherapy (rt), watch and wait protocol, general surgery, neoadjuvant radiation therapy, distal rectal cancer

## Abstract

The treatment of rectal cancer underwent a significant change with the introduction of total mesorectal excision (TME), which substantially improved recurrence rates. However, TME is associated with complications such as fecal incontinence and poor bladder control, especially in tumors located near the anal verge. The watch-and-wait (WW) protocol has emerged as an alternative for patients achieving a clinical complete response (cCR) following neoadjuvant radiochemotherapy. This narrative review, developed according to the Scale for the Assessment of Narrative Review Articles guidelines, evaluates neoadjuvant treatments and the WW protocol for rectal cancer. Literature was sourced from the PubMed database using specific search terms related to neoadjuvant therapy and the WW protocol, resulting in 63 articles selected for discussion. Neoadjuvant treatment, including chemoradiation and short-course radiotherapy, is indicated for T3 and T4 rectal adenocarcinomas. Studies like the German Rectal Cancer Study Group and the PRODIGE 23 trial have shown the benefits of preoperative treatment, including improved disease-free survival and reduced local recurrence rates. However, challenges in adopting the WW protocol include the risk of local regrowth and distant metastasis. Immune checkpoint inhibitors have shown promise in mismatch repair-deficient patients, yet the data are insufficient to fully endorse WW for these cases. The WW protocol is viable for selected rectal cancer patients, with ongoing debates regarding criteria for inclusion. Key challenges include accurately identifying cCR and managing patients with near-complete responses. MRI and endoscopic evaluation are crucial for assessing treatment response, although achieving a pathological complete response remains uncertain. The WW strategy offers a potential organ-preserving approach in rectal cancer management but requires careful patient selection and comprehensive risk-benefit discussions. Further research is needed to refine criteria for inclusion and optimize treatment protocols, enhancing outcomes while minimizing invasive interventions.

## Introduction and background

In 1982, a significant shift occurred in the treatment of rectal cancer with the introduction of the terms mesorectum and total mesorectal excision (TME) [1]. TME has been shown to reduce the recurrence rate of rectal cancer from 40% to less than 4%, making it a cornerstone procedure in modern rectal cancer surgery [1]. TME involves removing the mesorectum, which contains the loose connective tissue surrounding the rectum [1]. This procedure also includes the partial resection of local blood and nerve supplies, potentially leading to complications such as fecal incontinence and poor bladder control [2]. For tumors located less than 5 cm from the anal verge, preserving the anal sphincters and continence is rarely achievable, resulting in a low quality of life [2].

Habr-Gama et al. proposed the watch-and-wait (WW) protocol as an alternative to surgery for patients with T2-T4/N0-N2/M0 disease who have achieved a clinical complete response (cCR) after neoadjuvant radiochemotherapy (n-RCT) [2]. Observations indicate that overall survival (OS) and disease-free survival (DFS) in patients with cCR following n-RCT are comparable to those achieved with surgical TME [2]. In a follow-up study by the same author, which spanned over 10 years and included the initial patients treated with the WW protocol, most local regrowth occurred within the first year and could be easily detected through endoscopy. Most of these cases were successfully treated with salvage TME, achieving an impressive 94% DFS [3].

Two main conclusions emerged from this study: first, local regrowth likely results from failing to identify patients with a pathological complete response (pCR) during reassessment; second, most patients undergoing salvage TME had deep ypT3-T4 disease, which would not be detectable through traditional investigations such as digital rectal examination (DRE) or endoscopy. This study highlights the importance of accurately selecting patients for the WW protocol through thorough clinical evaluation and imaging [3].

For rectal adenocarcinomas treated with n-RCT or total neoadjuvant therapy (TNT), the complete pathological response rate ranges from 16% to 27% [4-6]. Observational studies with long follow-up periods suggest that WW in patients who achieve pCR after neoadjuvant therapy is associated with high rates of organ preservation and DFS comparable to those achieved with primary surgery. However, it is essential to consider that one-third of patients opting for surveillance may experience local recurrence [7,8]. Additionally, the number of patients meeting the criteria for this treatment strategy is relatively low.

The lack of clear standards for evaluating cCR presents challenges in designing randomized, prospective controlled trials. In 2014, the International Watch and Wait Database was established to address this issue and seek homogeneity [9]. One trial by Temmink et al. enrolled 1,010 patients from this database and concluded that, after a median follow-up of 2.9 years, the organ preservation rate was 79.3% (CI: 75.1-83.7%) [10]. Our review aims to describe the most current neoadjuvant therapy options in rectal cancer, including the WW strategy. Standard methods of evaluating oncological response and the effects of this strategy on patient outcomes with respect to DFS and OS are also detailed.

## Review

Selection criteria and analysis strategy

This review was developed following the Scale for the Assessment of Narrative Review Articles guidelines and is presented as a narrative review. Our analysis included literature on neoadjuvant treatment in rectal cancer and the WW protocol. We examined articles covering the following topics: approaches to neoadjuvant therapy, OS and DFS for patients in the WW protocol, criteria for including patients in the WW protocol, investigation methods and assessment periods for patients in the WW protocol, and local regrowth and metastasis rates for patients in the WW protocol compared to those who underwent TME. The studies were sourced from the PubMed database using the following search terms: “rectal neoadjuvant,” “watch and wait protocol,” “cCR in watch and wait,” and “pCR in watch and wait.” We identified 136 articles. Non-English language articles and case reports were excluded, resulting in 63 articles being selected for discussion. Two authors conducted the selection process to ensure impartial decisions. Disagreements were resolved by consultation with a third expert from another clinic.

Neoadjuvant therapy options and oncological outcomes in rectal cancer

The clearest indications with the best statistical evidence for neoadjuvant treatment are rectal adenocarcinomas at clinical stages T3 or T4. In these cases, chemoradiation or short-course radiotherapy is preferred before resection, followed by adjuvant treatment. Other debatable situations where neoadjuvant treatment can be considered include patients with clinically suspicious adenopathy on MRI or rectal echoendoscopy [11]. The data best illustrating the superiority of preoperative versus postoperative chemoradiotherapy comes from a study published in 2004 by the German Rectal Cancer Study Group. This study enrolled over 800 patients with T3 or T4 rectal adenocarcinoma or node-positive (N+) disease to receive either preoperative or postoperative radiotherapy [12]. The preoperative treatment consisted of 50.4 Gy, administered in fractions of 180 cGy per day, five days per week, along with radiosensitizing 5-fluorouracil (100 mg/m2) as a continuous infusion for 12 hours daily during weeks 1 and 5 of radiotherapy. The treatment regimen was similar in the group receiving postoperative radiotherapy, except that a boost was also used. OS was the primary endpoint.

Five-year OS was 76% in the preoperative treatment group, compared to 74% in the postoperative group (p = 0.8). The most significant difference was in the local recurrence rate, which clearly favored the preoperative treatment group (6% vs. 13%, p = 0.006). Late toxicity rates also favored preoperative treatment, with 14% versus 24% (p = 0.01) [12].

Given the analysis of the study, OS in non-metastatic rectal cancer depends more on the pathological response (yp) than on the initial clinical stage (c) [13,14]. Another important prognostic factor is tumor regression grade, which can be evaluated through several systems, including descriptions of the residual tumor and other related aspects such as fibrotic inflammation, the presence of acellular mucin, or calcification. Currently, the most used classification system is the American Joint Committee on Cancer Tumor Regression Grade [15].

TNT involves administering both oxaliplatin-based chemotherapy and radiochemotherapy preoperatively, with radiosensitizing fluoropyrimidine. Clear indications for this approach include stage T4 or N2 disease, tumors less than 5 cm from the anal verge, invaded mesorectal fascia, or extramural venous invasion [16]. Intensifying preoperative treatment in advanced rectal cancer aims to achieve the best possible pathological response, improving resectability and even organ preservation. Data supporting this approach come from the PRODIGE 23 trial, which enrolled patients with T3 or T4 rectal cancer and administered the FOLFIRINOX regimen for six cycles, long-course radiochemotherapy, followed by adjuvant chemotherapy with FOLFOX for three months in the experimental group, compared to the standard treatment with preoperative radiochemotherapy, surgical intervention, and six months of adjuvant treatment [17].

The study concludes that intensifying preoperative treatment significantly improved DFS and local recurrence rates compared to the standard alternative. Neurotoxicity was not a limiting factor in the more aggressive treatment. While PRODIGE 23 did not demonstrate an improvement in OS between the two groups at the three-year analysis, the data were more convincing at seven years (81.9% vs. 76.1%) [18]. Another noteworthy finding is that preoperative oxaliplatin use is associated with a lower incidence and severity of peripheral neuropathy than postoperative use [19].

The possible n-RCT regimens for advanced rectal cancer include conventional fractionation (long-course: total dose between 50.5 Gy and 54 Gy or short-course: 25 Gy in five fractions). The conventional regimen is preferred according to the phase 3 RAPIDO trial, demonstrating that locoregional relapses are more frequent with the short-course approach [20]. However, in the TNT approach, these two regimens were not compared. As neoadjuvant systemic therapy, FOLFIRINOX, FOLFOX, or CAPEOX can be used according to guidelines [17,21,22], with the latter two being less toxic alternatives for more fragile patients. It is important to note that the most robust data are from the PRODIGE 23 trial, and the chemotherapy triplet is preferable for all patients with Eastern Cooperative Oncology Group performance status 0-1 [23].

WW in mismatch repair deficient (dMMR) patients treated with neoadjuvant checkpoint inhibitors

Patients with dMMR or microsatellite instability-high (MSI-H) tumors are known to respond well to immune checkpoint inhibitors, regardless of the tumor location, making these agents a viable neoadjuvant therapeutic option. This approach can often lead to cCR and pCR, allowing for organ-sparing strategies [24]. However, only 3% of rectal cancers are dMMR/MSI-H [25]. Although these patients generally have a better prognosis, those with locally advanced tumors often exhibit reduced sensitivity to radiochemotherapy [26].

The most relevant data on using immune checkpoint inhibitors in neoadjuvant MSI-H locally advanced rectal cancer come from a prospective, phase 2 trial that enrolled 12 patients treated with dostarlimab every three weeks for six months. The trial continued radiochemotherapy and surgery for patients who did not achieve a complete clinical response. Patients were evaluated with DRE, pelvic MRI, 18F-fluorodeoxyglucose (FDG) PET, and rectoscopy with biopsy. At the time of publication, all 12 patients (100%, 95% CI: 74-100%) had achieved a complete clinical response and remained under follow-up, enabling organ preservation [26].

Additional data from the NICHE trial, which investigated anti-cytotoxic T-lymphocyte-associated protein-4 inhibitors combined with anti-programmed cell death protein-1 inhibitors before surgery in patients with early-stage colorectal cancer, are also noteworthy [27]. This trial included mismatch repair-proficient (pMMR, n = 20) and mismatch repair-deficient (dMMR, n = 40) patients. Before surgery, all participants received one dose of ipilimumab and nivolumab, followed by one dose of nivolumab with or without celecoxib. This study is significant because it provides a pathologically verified perspective on the efficacy of immune checkpoint inhibitors in the neoadjuvant setting. Among the dMMR patients, all showed a pathological response, with 19 having a major pathological response (MPR, defined as <10% remaining viable tumor tissue) and 11 achieving a complete pathological response. Among the pMMR patients, four of 15 showed a pathological response (three with MPR and one with a complete response). These findings can guide further studies in selecting colorectal cancer patients who may benefit from neoadjuvant immunotherapy [27].

Despite these promising results, the data are not yet sufficient to recommend a WW approach in patients with dMMR rectal cancer who received neoadjuvant immunotherapy and achieved a cCR. The WW protocol has not been widely adopted, partly due to uncertainty about oncological outcomes. OS can range from 100% at five years to 82% at three years, while DFS can be as high as 92% at five years and as low as 75% at three years (Table 1) [2,8,28-31].

**Table 1:** Summary of studies evaluating regrowth rates and survival outcomes in rectal cancer patients undergoing WW protocols DFS, disease-free survival; OS, overall survival; WW, watch and wait

Study (author and year)	Participants (n)	Study type	Reassessment time	Regrowth rate	OS	DFS
Habr-Gama et al. (2004) [2]	71	Observational	Eight weeks	2.80%	Five years: 100%	Five years: 92%
van de Valk et al. (2018) [8]	1,009	Observational	Multiple time intervals	25% at two years	Five years: 85%	Five years: 94%
Renehan et al. (2017) [28]	40	Observational	Eight weeks	34%	Three years: 95%	Three years: 88%
Garcia-Aguilar et al. (2022) [29]	105	Randomized	Four weeks, eight weeks, and 12 weeks	27%	Three years: 82%	Three years: 75%
van der Sande et al. (2019) [30]	385	Observational	-	23.10%	Two years: 98.4%	Two years: 90%
Wang et al. (2020) [31]	117	Case matched	-	14.90%	-	Three years: 88%

The considerable heterogeneity in published data often leads to non-standardized procedures. The most pressing issue is the local relapse or regrowth rate, which can occur in up to 34% of cases, as demonstrated by Renehan et al. [28], but can be as low as 2.8%, according to Habr-Gama et al. (Table 1) [2]. In cases of local regrowth, salvage surgery and TME are possible in up to 90% of cases. In select cases, early identification allows for local resection (transanal or endoscopic) as an alternative to salvage TME with similar recurrence rates [32]. In patients with complete clinical response, local recurrence usually occurs within the first two to three years [33]. Although local tumor control is important, the WW protocol has another drawback: patients under the WW protocol report higher rates of distant metastasis than those undergoing TME, reaching up to 18%. These conclusions were reported by van der Valk et al. in a multicenter trial [8] and Smith et al. in a study concerning patients in the WW protocol [34].

Tumor traits and patient inclusion in the WW protocol

The initial patients enrolled in the WW protocol were selected based on the outcome of neoadjuvant therapy, specifically the achievement of a cCR, rather than pretreatment clinical characteristics. A patient is considered to have a cCR if no macroscopic residual tumor is identifiable through clinical investigations [34]. Initial paraclinical evaluation of rectal cancer patients should follow the European Society for Medical Oncology guidelines, which include DRE, CT of the thorax and abdomen, pelvic MRI, colonoscopy, and histopathology report [35].

Currently, there are no standard criteria for including patients in the WW protocol. We recommend the criteria used by the authors of the Organ Preservation in Rectal Adenocarcinoma (OPRA) trial: age over 18 years, T3-T4/N0 stage, confirmed histopathological diagnosis of rectal adenocarcinoma, and no distant metastasis. The OPRA trial is unique as it is one of the few prospective studies on this subject and offers a three-tiered regression schema to allow physicians to evaluate the response objectively.

Habr-Gama et al.’s landmark trial emphasizes that the tools or investigative methods used in the initial evaluation to include the patient in the WW protocol must also be available at reassessment. For these candidates, it is important to observe and note the pretreatment endoscopic, clinical, and radiological traits of the tumor to identify post-treatment patients with complete, near-complete, or incomplete responses [2,36]. Tumor location in relation to the anal sphincter is crucial when selecting this therapy type. Tumors should be located no more than 7 cm above the anal verge [37].

This 7-cm cutoff is based on the risk-benefit balance. Tumors above this threshold, when treated with TME, have a lower risk of poor functional outcomes and stoma creation. DRE should be used to evaluate the tumor location in all candidates for the WW protocol. MRI can be a standalone tool for patient stratification and can identify tumor characteristics that may predict local or distant failure. DRE during the first assessment and reassessment should be done by the same physician.

If the tumor is located at or below the level of the levator ani pelvic diaphragm, the patient can be a candidate for the WW protocol. In contrast, tumors with a distal edge more than 1 cm above this level should be considered for TME [37,38]. The WW protocol should not be applied to tumors extending the entire length of the rectum due to increased relapse rates, as shown in Table 2 [31,39-41].

**Table 2:** Comparison of local recurrence rates in rectal cancer patients treated with WW versus TME TME, total mesorectal excision; WW, watch and wait

Study (author and year)	Patients (n)		Incidence of local recurrence	
	WW	TME	WW	TME
Wang et al. (2020) [31]	59	179	11.90%	0.50%
Ayloor Seshadri et al. (2013) [39]	23	10	30.40%	0%
Araujo et al. (2015) [40]	42	69	19%	4,7%
Yeom et al. (2019) [41]	15	129	11%	0%

Mesorectal fascia penetration or high-risk tumor characteristics such as high-grade adenocarcinoma or mucinous histopathology should not exclude patients from the WW protocol if a cCR is obtained at reassessment after neoadjuvant radiotherapy [37,42]. However, patients with circumferential margin invasion greater than 1 mm or venous dissemination away from the tumor are associated with lower cCR rates and should be excluded [43,44]. Patients with enlarged lymph nodes in the mesorectum, lateral pelvic sidewall, or venous invasion outside the radiotherapy field should be excluded from the WW protocol even if cCR is obtained [37,42]. Large ulcerated or circumferential tumors should be closely monitored due to the risk of post-therapy scarring and local stenosis [2]. Sequence variation in genes such as TP53 or KRAS can make tumors resistant to neoadjuvant therapy and decrease cCR rates [45,46].

Evaluating treatment response

cCR does not guarantee a pCR. Investigation methods such as MRI, CT, or PET lack the sensitivity to identify pCR reliably. The ideal method for identifying patients with pCR remains debated. MRI should be the preferred method, but it needs to be correlated with endoscopic evaluation and DRE, as per the European Society of Gastrointestinal and Abdominal Radiology (ESGAR) consensus, to maximize results [47]. High-resolution MRI is crucial for the initial evaluation and staging of rectal cancer. It can anatomically distinguish the rectal wall from the mesorectal fascia, differentiating between locally advanced and localized tumors. MRI can also detect prognostic factors such as vascular invasion, particularly venous, peritoneal spread, or extramural bowel invasion [48].

de Jong et al. evaluated the response to radiochemotherapy using MRI and compared the results with postoperative histopathological evaluation of the specimens [44]. They found that MRI has 75% accuracy in identifying cCR, 31% specificity, and 47% ability to predict negative cCR. The study reported a 95% sensitivity in identifying patients with incomplete cCR and an 83% predictive ability [44]. Based on these findings, MRI could successfully rule out cCR. The MERCURY trial demonstrated that tumor regression post-neoadjuvant chemoradiotherapy (nCRT), confirmed by MRI, correlates with DFS, OS, and local recurrence rate [42]. To compare pre- and post-nCRT MRI images, consistent acquisition and interpretation parameters for clinical staging and post-nCRT are necessary, as provided by the ESGAR and Society of Abdominal Radiology [47,49].

Maas et al. recommended using MRI with DRE and endoscopy, achieving up to 98% accuracy in predicting cCR [50]. However, even when all three methods indicated the presence of residual tumors, 15% of these patients still achieved pCR [50].

The time to reassessment when evaluating the tumor response after radiochemotherapy is important. Longer intervals between treatment completion and surgery are associated with higher pCR rates. Gambacorta et al. conducted a meta-analysis of over 3,000 patients across seven trials, finding that up to 95% of patients with pCR were operated on after 10 weeks of completing the last treatment cycle [51]. Thus, a standard interval of eight to 10 weeks for reassessment after radiochemotherapy should be adopted [51]. This strategy aims to increase pCR rates after nCRT and surgery. The data are also supported by the TIMMING study [52]. Another way to evaluate the treatment response is 18F-FDG PET, which can assess the functional status of the residual tumor tissue. Crimì et al. found a direct correlation (p = 0.037) between the intensity of 18F-FDG uptake and local response, indicating that more residual tumor tissue results in more uptake [53]. This functional information from PET can be combined with MRI’s morphological information for a comprehensive view of the tumor [53].

In a systematic review by Maffione et al., PET-CT demonstrated a predictive sensitivity of 79% and a specificity of 78% for early evaluation of treatment response [54]. However, a significant limitation of PET-CT is the increased uptake of the radiotracer, primarily related to chronic inflammation generated by radiotherapy [55]. This uptake is also limited in lymph nodes due to their small size. Consequently, PET-CT is not recommended as a routine investigation but can be used with MRI.

Although assessing local response to n-RCT is important, up to 16% of patients with cCR are diagnosed with lymph node metastasis in the pelvic sidewall or mesorectum, as reported by Park et al., Kim et al., and Kim et al. [14,56,57]. Non-regional lymphatic nodes located around the external iliac artery in cCR patients have double the risk of distant metastasis without an increase in local recurrence rates [58]. Currently, no consensus exists on the optimal criteria to identify and define metastatic lymph nodes in rectal cancer from an imaging and functional perspective [59].

Lymph nodes are considered suspicious if they are enlarged to over 1 cm on MRI and have at least two of the following morphologic characteristics: irregular margins, round shape, or variable signal intensity. Indirect characteristics of the primary tumor may also suggest lymphatic dissemination. Custers et al. found a direct correlation between tumor depth and lymph node involvement [60]. Correlating local tumor response with nodal invasion requires further research [60].

Another consideration when evaluating the treatment response involves patients with near cCR, defined as having a good response at the initial evaluation but lacking the criteria for cCR on endoscopy or MRI. To study cCR and near cCR, Temmink et al. compared over 1,000 patients, dividing them into two groups: one with complete cCR and one with near-complete cCR [10]. They used MRI and endoscopy for stratification. Initially, 608 patients achieved cCR, while 402 achieved cCR at a later reassessment, with a median follow-up of 2.6 years for the first group and 2.9 years for the second. There was no difference in DFS and OS.

MRI evaluation showed a better organ preservation rate in the near cCR group. Extending the observation period for this special category of patients can lead to cCR. Advanced T3 and T4 tumors represent the bulk of patients with near cCR. Locally invasive tumors take longer to achieve cCR but can be achieved and sustained. If there is no improvement in the local tumor response upon reassessment, the WW protocol should be abandoned, and the patient should undergo salvage TME. Temmink et al. also noted that a large proportion of patients with near cCR received chemotherapy within six months after evaluation, increasing the likelihood of achieving cCR [10]. This observation aligns with the RAPIDO [20] and OPRA trials [21], which noted that chemotherapy can increase the rate of cCR after radiotherapy, especially in the case of near cCR.

In the Stockholm III study, patients who did not receive chemotherapy had a pCR rate of only 10%, compared to 28% in the RAPIDO trial and nearly 50% in the OPRA trial [20,21,61]. Patients with a near-complete response should be given an additional four to eight weeks to progress to cCR.

Impact of WW strategies on survival

Several studies aim to compare survival outcomes in patients with advanced rectal cancer who received TNT followed by follow-up versus those who received TNT followed by surgery. A study published in 2022 by Garcia-Aguilar et al. compared patients who underwent TNT followed by a WW approach with resection at recurrence to those who received TNT and TME as primary treatment. The study demonstrated that DFS was similar in both cohorts [29].

According to current studies, the vast majority of relapses during WW occur in the first two years [21,62]. The OPRA study demonstrates that the preservation of the rectum is possible in more than half of the cases for which WW is chosen and that there is no detrimental effect on distant metastasis-free survival or OS. DFS was similar in this study for patients who underwent surgery after re-staging versus those who received surgery after regrowth [21]. However, there are observational studies that demonstrate that the conservative option is associated with a higher rate of distant metastasis or death [62,63].

## Conclusions

Currently, in rectal cancer, there are numerous neoadjuvant treatment options, especially in advanced cases, ranging from radiochemotherapy to TNT to treatment with immune checkpoint inhibitors. Because the response to neoadjuvant therapy is one of the most important prognostic factors, choosing the correct treatment is essential. In patients who have a complete clinical response, the use of a WW protocol can be a preferred option to surgery, as the latter has numerous initial and late complications. The WW protocol consists of careful surveillance involving rectosigmoidoscopy and imaging at short intervals (three to six months), which may seem burdensome to the patient. This should be considered especially for patients with tumors located close to the anus or in patients prone to postoperative complications. The risks and benefits of this method must be discussed in detail with the patients.
